# Supplementary material for: Extending PROXIMAL to predict degradation pathways of phenolic compounds in the human gut microbiota
Source: NPJ Syst Biol Appl. 2024 May 27;10:56. doi: 10.1038/s41540-024-00381-1 (PMC11130242; doi:10.1038/s41540-024-00381-1)
Supplement: Supplementary file 1 — Supplementary Information [file 41540_2024_381_MOESM1_ESM.pdf]

Supplementary Information: PROXIMAL2: a new enzyme promiscuity method for complex multi-step reactions

Francesco Balzerani[1], Telmo Blasco[1], Sergio Pérez-Burillo[1], Luis Valcarcel[1,2,3], Francisco J. Planes[1,2,3*] and Soha Hassoun[4,5*]

[1]University of Navarra, Tecnun School of Engineering, Manuel de Lardizábal 13, 20018 San Sebastián, Spain.

[2]University of Navarra, Biomedical Engineering Center, Campus Universitario 31009 Pamplona, Navarra, Spain.

[3]University of Navarra, Instituto de Ciencia de los Datos e Inteligencia Artificial (DATAI), Campus Universitario, 31080, Pamplona, Spain

[4]Department of Computer Science, Tufts University, Medford MA, 02155, USA

[5]Department of Chemical and Biological Engineering, Tufts University, Medford MA, 02155, USA

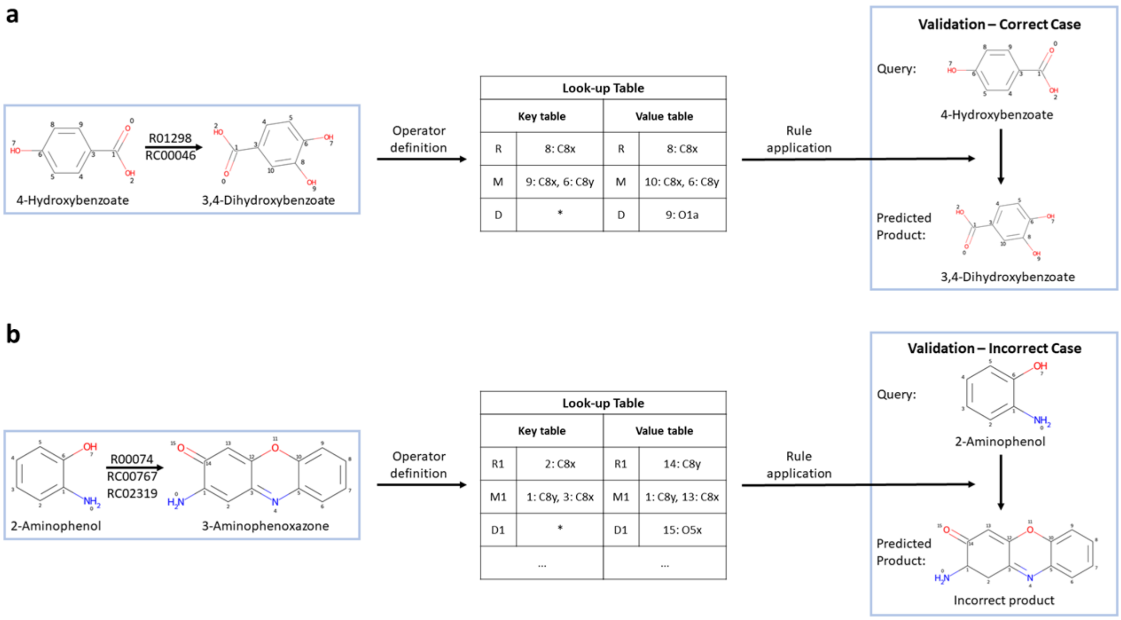

Supplementary Figure 1: Validation strategy of PROXIMAL2. The validation strategy of PROXIMAL2 consists of generating the key and value tables for every substrate (S)-product (P) pair, following the methodology presented in the Methods section, and then assess if the product P is obtained when the same substrate S is applied as a query compound to their associated key and value tables. Rules (key and value tables) that satisfy this requirement correctly captures the underlying chemistry of the associated reaction. Here, we present one positive and negative case. We used this validation strategy to compare PROXIMAL and PROXIMAL2, finding a statistically significant over-performance of PROXIMAL2, as it is detailed in Figure 1 in the main text. **a)** Example reaction where PROXIMAL2 correctly generates the annotated product (*3,4-Hydrobenzoate*) when the annotated substrate (*3-Hydrobenzoate*) is applied as a query compound of their corresponding reaction rule defined in the look-up table; **b)** Example reaction where PROXIMAL2 does not recover the annotated product (*3-Aminophenoxazone*) when the annotated substrate (*2-Aminophenol*) is applied as a query compound of their corresponding reaction rule defined in the look-up table.

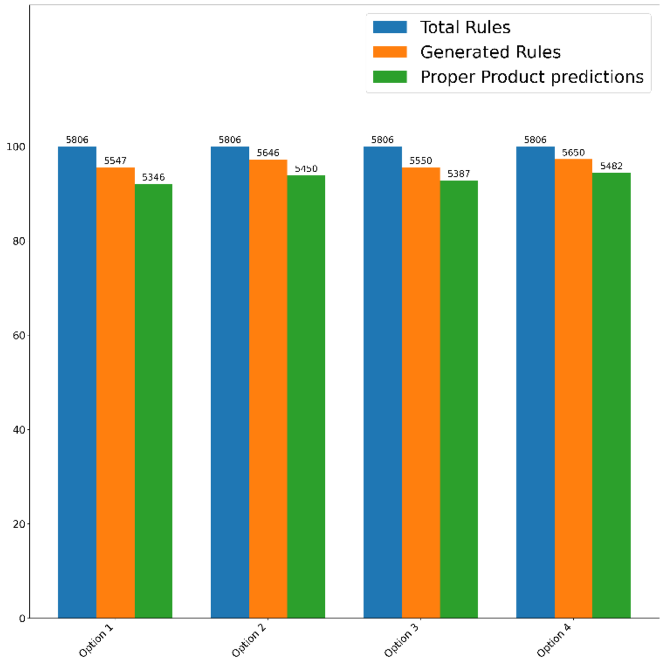

Supplementary Figure 2: Sensitivity analysis of PROXIMAL2. In the construction of the Maximum Common Substructure (MCS) in PROXIMAL2 we fixed 2 optional parameters: '*ringMatchesRingOnly*' and '*match_Valence*'. We carried out a sensitivity analysis to evaluate the effect of these heuristic choices in the outcome of PROXIMAL2. In particular, we considered the 4 possible cases, namely when both parameters are fixed, the two cases where only one of the parameters is fixed and none of the parameters are fixed. Here, we show that best performance is obtained in the case that both parameters are considered, which justifies the use of these parameters in PROXIMAL2. In particular, we present a barplot representing the reaction coverage with look-up tables and those correctly predicting the annotated product in KEGG reactions under 4 scenarios: both *ringMatchesRingOnly* and *match_Valence* are set FALSE (Option 1), *ringMatchesRingOnly* is set TRUE and *match_Valence* FALSE (Option 2), *ringMatchesRingOnly* is set FALSE and *match_Valence* TRUE (Option 3) and both *ringMatchesRingOnly* and *match_Valence* are set TRUE (Option 4). The y-axis shows the coverage in percentage. The total number of reactions are indicated over the bars.

a

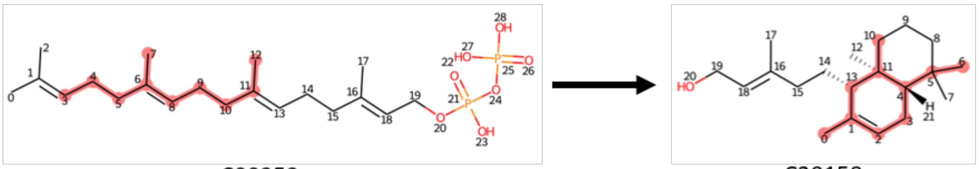

C00353 C20158

Alignment via MCS	C00353	3	4	5	6	7	8	9	10	11	12
	C20158	10	11	13	1	0	2	3	4	5	6

b

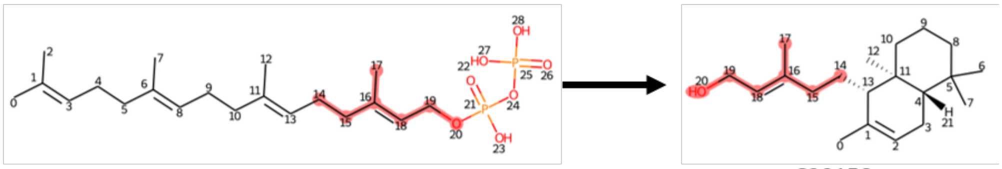

C00353 C20158

Alignment via MCS	C00353	14	15	16	17	18	19	20
	C20158	14	15	16	17	18	19	20

c

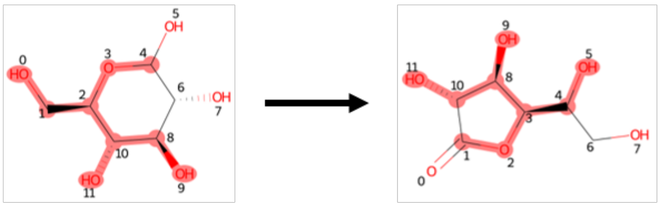

C00031 C03107

Alignment via MCS	C00031	0	1	2	3	4	8	9	10	11
	C03107	5	4	3	2	1	10	11	8	9

d

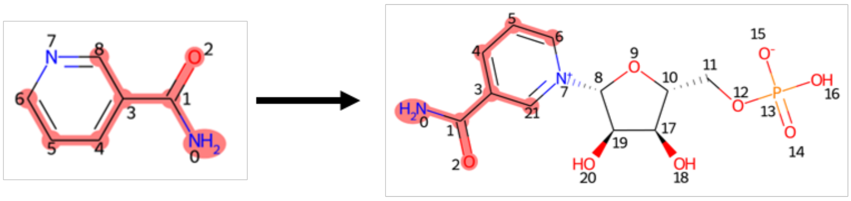

C00153 C00455

Alignment via MCS	C00153	0	1	2	3	4	5	6	8
	C00455	0	1	2	3	4	5	6	21

Supplementary Figure 3: Illustration of different parameters in the maximum common substructure (MCS) algorithm. **a)** Resulting MCS for the KEGG reaction *R09874* with the parameter *ringMatchesRingOnly* inactive; **b)** Resulting MCS for the KEGG reaction *R09874* with the parameter *ringMatchesRingOnly* active; **c)** Representation of the partial match between subgraph of the rings in the substrate and product due to the parameter *ringMatchesRingOnly*. KEGG reaction id: *R00301*); **d)** Resulting MCS with the parameter *matchValence* active, which discards matching between atoms with a different valence. KEGG reaction: *R00828*. In all the cases MCS is shown in red colour.

a

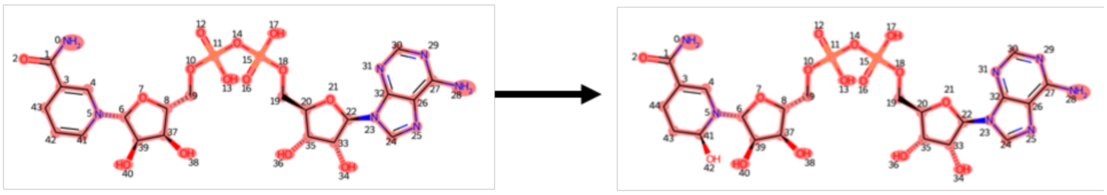

C00004 C04856

Alignment via MCS	C00004	0	1	2	3	…	40	41	42	43
	C04856	0	1	2	3	…	40	41	43	44

b

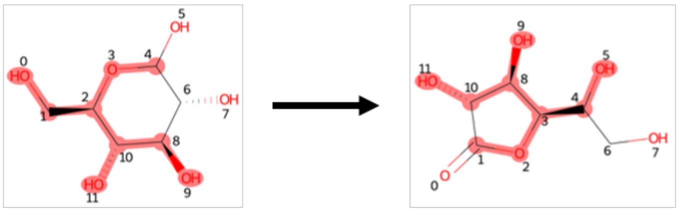

C00031 C03107

Alignment via MCS	C00031	0	1	2	3	4	8	9	10	11
	C03107	5	4	3	2	1	10	11	8	9

Supplementary Figure 4: Representation of bond changes between atoms belonging to the MCS. In multi-step reactions the substrate goes through several structural transformations, as reflected in Step 4 in the PROXIMAL2 pipeline. In some cases, structural changes can involve bonds between atoms belonging to the Maximum Common Substructure, which is not reflected in standard 'key' tables. Changes include the order of a bond a removal/formation of a bond. In order to consider these changes in the look-up tables, the bonds between atoms included in the MCS are analysed and compared between the substrate and the product, checking for differences and modifications. Then, similarly to reaction centres, the atoms involved in the bonds and their respective adjacent and distant neighbours are extracted. **a)** Definition of the MCS for the KEGG reaction *R00129.* The bond between atom 41 and 42 in the substrate (C00004) is a double bond and the corresponding bond between atom 41 and 43 within the product (C04856) is a single bond due to the addition of the functional group. KEGG (Kanehisa and Goto, 2000) reaction: *R00129*. The MCS is not completely included. However, from atom 0 to atom 41 the alignment is numerically exact in the two molecules; **b)** Resulting MCS for the KEGG reaction *R00301*. The bond between atom 10 and 1 in the product (*C03107*) is not present on the substrate (C00031), whose corresponding atoms are number 4 and 8. This is a multi-step reaction, where, in addition to other structural changes, it can be observed the hexagon ring cleavage and the formation of the pentagon ring within the MCS.

Supplementary Note 1: Selected parameters in the Maximum Common Substructure algorithm in RDKit

In the step 3 of the PROXIMAL2 pipeline, detailed in the main text, substrate-product pairs are aligned with the maximum common substructure (MCS) algorithm that is available in RDKit (Landrum, 2011): function *findMCS* in the module *rdFMCS*. We made use of 3 specific parameters:

- *bondCompare*. It is defined as *CompareOrderExact*, which ensures that the common substructures share the same bond type and order;

- *ringMatchesRingOnly*. It is defined as *True* and it guarantees that atoms belonging to rings in substrates match only with atoms belonging to rings in products. Supplementary Figure 3a shows an example MCS where this parameter is not considered, leading to a meaningless match between atoms in a linear structure in the substrate, C00353, and atoms belonging to a ring in the product, C20158. This issue is corrected in Supplementary Figure 3b, where this parameter is introduced. Moreover, the parameter provides the capacity of the algorithm to extract the partial match between rings, as shown in Supplementary Figure 3c, where the alignment between atoms of the rings is not complete;

- *matchValence*. This parameter is set as *True* and it considers the valence of the atoms in the MCS, discarding a match between atoms with a different valence. Supplementary Figure 3d shows a case when the difference in valence of the atoms determines the partial match of the substructures. In particular, the atom 1 in the substrate, C00019, and atom 14 in the product, C00021, are the same atom, but they differ in valence and, therefore, the correspondence is denied.

References

Kanehisa,M. and Goto,S. (2000) KEGG: Kyoto Encyclopedia of Genes and Genomes. *Nucleic Acids Res.*, **28**, 27–30.

Landrum,G. (2011) RDKit : A software suite for cheminformatics , computational chemistry , and predictive modeling. *Components*.
